# SEOM SOGUG clinical guideline for treatment of kidney cancer (2022)

**DOI:** 10.1007/s12094-023-03276-5

**Published:** 2023-08-09

**Authors:** María José Méndez-Vidal, Martin Lázaro Quintela, Nuria Lainez-Milagro, Begoña Perez-Valderrama, Cristina Suárez Rodriguez, José Ángel Arranz Arija, Ignacio Peláez Fernández, Enrique Gallardo Díaz, Julio Lambea Sorrosal, Aránzazu González-del-Alba

**Affiliations:** 1 Medical Oncology Department, Maimonides Institute for Biomedical Research of Cordoba (IMIBIC), Hospital Universitario Reina Sofía, Córdoba, Spain; 2 Medical Oncology Department, Hospital Alvaro Cunqueiro-Complejo Hospitalario Universitario de Vigo, Pontevedra, Spain; 3 Medical Oncology Department, Hospital Universitario de Navarra (HUN), Pamplona, Spain; 4 Medical Oncology Department, Hospital Universitario Virgen del Rocío, Seville, Spain; 5 Medical Oncology Department, Hospital Universitario Vall D’Hebron, Barcelona, Spain; 6 Medical Oncology Department, Hospital General Universitario Gregorio Marañón, Madrid, Spain; 7 Medical Oncology Department, Hospital de Cabueñes, Gijón, Spain; 8 Medical Oncology Department, Corporació Sanitària Parc Taulì, Barcelona, Spain; 9 Medical Oncology Department, Hospital Clínico Universitario Lozano Blesa, Zaragoza, Spain; 10 Medical Oncology Department, Hospital Universitario Puerta de Hierro-Majadahonda, Madrid, Spain

**Keywords:** Renal cell carcinoma, Diagnosis, Staging, Management, Systemic therapy, Guidelines

## Abstract

Renal cancer is the seventh most common cancer in men and the tenth in women. The aim of this article is to review the diagnosis, treatment, and follow-up of renal carcinoma accompanied by recommendations with new evidence and treatment algorithms. A new pathologic classification of RCC by the World Health Organization (WHO) was published in 2022 and this classification would be considered a “bridge” to a future molecular classification. For patients with localized disease, surgery is the treatment of choice with nephron-sparing surgery recommended when feasible. Adjuvant treatment with pembrolizumab is an option for intermediate-or high-risk cases, as well as patients after complete resection of metastatic disease. More data are needed in the future, including positive overall survival data. Clinical prognostic classification, preferably IMDC, should be used for treatment decision making in mRCC. Cytoreductive nephrectomy should not be deemed mandatory in individuals with intermediate-poor IMDC/MSKCC risk who require systemic therapy. Metastasectomy can be contemplated in selected subjects with a limited number of metastases or long metachronous disease-free interval. For the population of patients with metastatic ccRCC as a whole, the combination of pembrolizumab–axitinib, nivolumab–cabozantinib, or pembrolizumab–lenvatinib can be considered as the first option based on the benefit obtained in OS versus sunitinib. In cases that have an intermediate IMDC and poor prognosis, the combination of ipilimumab and nivolumab has demonstrated superior OS compared to sunitinib. As for individuals with advanced RCC previously treated with one or two antiangiogenic tyrosine-kinase inhibitors, nivolumab and cabozantinib are the options of choice. When there is progression following initial immunotherapy-based treatment, we recommend treatment with an antiangiogenic tyrosine-kinase inhibitor. While no clear sequence can be advocated, medical oncologists and patients should be aware of the recent advances and new strategies that improve survival and quality of life in the setting of metastatic RC.

## Introduction

Renal cell carcinoma (RCC) constitutes 80% of all primary renal neoplasms. The incidence remains stable in recent years with 431,288 new cases and 179,368 deaths in 2020 according to data published by GLOBOCAN. In Spain, the estimated incidence in 2022 was 8078 new cases (5572 in men and 2506 in women). It is twice as common in males and the median age at diagnosis is 64 years. Known risk factors include smoking, obesity, and hypertension. It is more prevalent among people with chronic renal failure, dialysis, renal transplant recipients, or those with tuberous sclerosis. Two percent are hereditary, normally Von Hippel-Lindau disease. Patients with bilateral tumors or characteristic alterations should be tested for germline mutations [1–3]

## Diagnosis

Most tumors of the kidney (60%) were diagnosed incidentally by radiologic procedures performed for other medical indications [4]. The classic triad of flank pain, visible haematuria, and palpable abdominal mass is rare (6–10%) and correlates with aggressive histology, advanced disease, and worse outcomes [5]. Nonetheless, RCC is still the “internist’s cancer,” with paraneoplastic syndromes, such as hypercalcemia, unexplained fever, or erythrocytosis in approximately 30% of all cases [6]. Germline mutation testing and genetic counseling should be contemplated for younger patients (≤ 46 years) with RCC [7].

A physical examination should be performed together with a complete medical history. Laboratory tests include complete blood cell count, lactate dehydrogenase (LDH), serum creatinine, liver function study, serum-corrected calcium, and urinalysis [8].

Diagnosis is typically suggested by abdominal ultrasound, albeit an abdominal computed tomography scan (CT) is the gold standard to assess local invasiveness, venous involvement, locoregional lymph node involvement, or distant metastases. Nevertheless, it reveals poor differentiation between solid masses, fat-poor angiomyolipoma, and oncocytoma [9]. CT sensitivity for small renal masses surpasses 90%, approaching 100% for lesions > 2 cm [10]. In the case of a solid renal mass, the key criterion for malignant lesions is the presence of contrast enhancement or restriction. CT perfusion imaging detects temporal changes in tissue attenuation. It can pick up changes at the molecular level and evaluate tissue perfusion and vascular permeability. Perfusion studies are an indirect predictor of neoangiogenesis [11] and sensitivity and specificity to predict RCC were 100%, and 66.7%, respectively [12]. A chest CT is recommended, except in cT1a renal tumors, for which the probability of a positive chest CT is low [13].

Magnetic resonance imaging (MRI) is useful when evaluating local invasion and inferior vena cava involvement is suspected, or in case of allergy to the CT contrast agent or renal insufficiency. Routine bone or brain imaging is not indicated [14]. Bone scans can be performed if the subject has elevated serum alkaline phosphatase or reports bone pain. Cerebral CT or MRI will be carried out if clinical signs and symptoms point to brain metastases.

Fluorodeoxyglucose (FDG) Positron Emission Tomography (PET) is not currently regarded to be the standard imaging modality for the diagnosis of renal cancer [15], given its low sensitivity. As FDG is excreted by the kidneys, FDG PET is not suitable for local staging of primary RCC, but can be useful in metastatic RCC and to evaluate response to therapy. Although FDG is the most widely used tracer for PET scans, other new tracers are being studied, such as 68 Ga-PSMA, 18Ffluoroethylcholine,11C-acetate, 18F-fluoromisonidazole, and 18F-fluorothymidine [16, 17]. A renal tumor core biopsy provides high sensitivity (86–100%) and specificity (98–100%) to histopathologically confirm malignancy [18]. Needle core biopsies are preferable over fine needle aspiration for solid renal masses (RMs) [19], and is especially recommended prior to ablative therapies, as well as in patients with advanced disease before initiating systemic treatment [20].

## Staging

The 2017 TNM classification is the recommended clinical staging standard [21, 22] (Tables 1 and 2).

**Table 1:** Kidney cancer TNM-staging AJCC UICC 2017

Stage		Definition		Subdivision
Tumour stage				
Tx	Primary tumour cannot be assessed			
T0	No evidence of primary tumour			
T1	Tumour 7 cm or less in greatest dimension, limited to the kidney			**T1a:** ≤ 4 cm **T1b:** > 4 cm but < 7 cm
T2	Tumour more than 7 cm in greatest dimension, limited to the kidney			**T2a:** > 7 cm but < 10 cm **T2b:** > 10 cm
T3	Tumour extends into major veins or perinephric tissues but not into the ipsilateral adrenal gland and not beyond Gerota fascia			**T3a:** Tumour extends into the renal vein or its segmental branches, or tumour invades the pelvicalyceal system or tumour invades perirenal and/or renal sinus fat (peripelvic) fat but not beyond Gerota fascia **T3b**: Tumour extends into vena cava below diaphragm **T3c:** Tumour extends into vena cava above the diaphragm or invades the wall of the vena cava
T4	Tumour invades beyond Gerota fascia (including contiguous extension into the ipsilateral adrenal gland)			
Regional lymph nodes				
Nx	Regional lymph nodes cannot be assessed			
N0	No regional lymph node metastasis			
N1	Metastasis in regional lymph node(s)			
Distant metastasis				
M0	No distant metastasis			
M1	Distant metastasis

**Table 2:** Stage grouping for RCC based on AJCC TNM 2017

Stage			
Stage I	T1	N0	M0
Stage II	T2	N0	M0
Stage III	T3 T1, T2, T3	N0	M0
		N1	M0
Stage IV	T4	Any N	M0
	Any T	Any N	M1

## Recommendations

- CT scan is the gold standard for RCC staging. Level of evidence: III. Grade of recommendation: A.
- Abdominal MRI is an alternative in various circumstances. Level of evidence: III. Grade of recommendation: C.
- Neither bone scans nor brain CT (nor MRI) are recommended for routine clinical practice. Level of evidence: III. Grade of recommendation: D.
- In patients without previous tumor diagnosis, a renal tumor core biopsy is recommended before treatment with ablative therapies, as well as in cases of metastatic disease, prior to starting systemic treatment. Level of evidence: III. Grade of recommendation: A.

## Pathological and molecular classification

Renal cell carcinoma (RCC) is a heterogeneous disease. Histological classification is based on the tumor origin from different cells located in nephron cells but is completed with molecular and clinical information. The most common type of RCC is clear-cell renal cell carcinoma (ccRCC), accounting for up to 75% of all RCCs. Other major types include papillary (10–15%), chromophobe (5%), oncocytic (< 5%), Xp11 translocation (< 1%), or collecting-duct carcinomas (< 1%) [23]. There is a new pathology classification of RCC by the World Health Organization (WHO), 5th edition, published in 2022 (Table 3). We are witnessing an important change in papillary RCC pathological classification. No distinction is made between subtypes 1 and 2. Molecular research suggests that type 2 papillary RCC may not, in fact, constitute a truly independent entity [24]. New molecularly defined RCC subtypes are named and described: Eosinophilic solid and cystic RCC, elonging C (ELOC)-mutated RCC (formerly denominated Transcription elongation factor B (TCEB1)-mutated RCC), ALK-rearranged RCC, SMARCB1-deficient medullary RCC, TFEB-altered RCC, and fumarate hydratase (FH)-deficient RCC (formerly hereditary leiomyomatosis (HLRCC) syndrome-associated RCC. This classification “bridges” the present to a future molecular classification. Some entities are now regarded as independent types of RCC with specific clinical and molecular features, such as sporadic FH-deficient RCC, tubulocystic RCC, ESC RCC, clear cell papillary RCC, SMARCB1-deficient RCC, and microphthalmia-associated transcription factor (MiTF) family RCC [25].

**Table 3:** WHO 2022 Classification of renal cell tumors

Clear cell renal tumors
Clear cell renal cell carcinoma
Multilocular cystic renal neoplasm of low malignant potential
Papillary renal tumors
Papillary adenoma
Papillary renal cell carcinoma
Oncocytic and chromophobe renal tumors
Oncocytoma
Chromophobe renal cell carcinoma
Other oncocytic tumors of the kidney
Collecting duct tumors
Collecting duct carcinoma
Other renal tumors
Clear cell papillary renal cell tumor
Mucinous tubular and spindle cell carcinoma
Tubulocystic renal cell carcinoma
Acquired cystic disease-associated renal cell carcinoma
Eosinophilic solid and cystic RCC
Renal cell carcinoma, NOS
Molecularly defined renal carcinoma
* TFE3*-rearranged renal cell carcinoma
* TFEB*-altered renal cell carcinoma
ELOC (formerly *TCEB1-*) mutated renal cell carcinoma
Fumarate hydratase-deficient renal cell carcinoma
Hereditary leiomyomatosis and renal cell carcinoma syndrome-associated renal cell carcinoma
Succinate dehydrogenase-deficient renal cell carcinoma
* ALK* rearrenged renal cell carcinoma
Medullary carcinoma, NOS
* SMARCB1*-deficient medullary-like renal cell carcinoma
* SMARCB1*-deficient undifferentiated renal cell carcinoma, NOS
* SMARCB1*-deficient undifferentiated renal cell carcinoma of other specific subtypes

Papillary RCC is considered the classical papillary RCC morphology type I, albeit certain single entities with papillary features can be regarded as variants of papillary RCC or provisional entities like papillary renal neoplasm with reversed polarity (PRNRP), biphasic hyalinizing psammomatous RCC (BHP RCC), biphasic squamoid/alveolar RCC, or thyroid-like follicular RCC (TLF RCC) Some have a specific molecular driver alteration, for example, KRAS mutations in PRNRP, NF2 mutations in BHP RCC, and EWSR1-PATZ1 fusions in TLF RCC.

While certain entities with eosinophilic or oncocytic cytoplasm are well defined, such as SDH-deficient RCC, ESC RCC, and FH-deficient RCC, others are emerging entities, for instance, eosinophilic vacuolated tumor (EVT) and low-grade oncocytic tumor (LOT). TSC mutations are important and are common in ESC RCC; similarly, TSC1/2 mutations or activating mTOR mutations have also been identified in EVT and LOT [25].

The differential diagnosis between cromophobe RCC and oncocytoma is essential. Chromophobe RCCs exhibit diffuse positivity for cytokeratin 7 (CK7), whereas oncocytomas are negative or present focal positivity for CK7. Molecular studies provide a more definitive diagnosis of subtypes.

ChRCC is characterized by chromosomal aneuploidy, TP53, PTEN, and mitochondrial gene mutations [26]. Collecting duct carcinoma (CDC), or Bellini duct carcinoma, continues to be a highly aggressive RCC arising from the renal collecting tubules or distal convoluted tubule. Recent NGS research has highlighted genomic alterations in SETD2, CDKN2A, SMARCB1, and NF2, and, together with transcriptomic studies, has confirmed the differences between urothelial carcinoma and CDC. Moreover, these tumors are characterized by having enriched tumor-infiltrating lymphocytes [27].

Sarcomatoid features are present in < 10% of RCC tumor and are mainly observed in patients with predominant clear cell areas [28]. Mutations in the gene encoding von Hippel–Lindau (VHL) disease tumor suppressor that leads to stabilization of hypoxia-inducible factor (HIF) are present in most ccRCCs (sporadic and hereditary forms). Loss of VHL function results in an upregulation of angiogenesis [29]. The Cancer Genome Atlas (TCGA) performed a comprehensive analysis of more than 400 ccRCC tumors that exhibited other mutated genes. The VHL gene was mutated in nearly 90% of the patients, while mutations that modify the chromatin-remodeling complex (PBRM1, ARID1A, and SMARCA4) and other epigenetic regulators, such as SETD2 and BAP1, are also commonly found [29, 30]. Although distinct histology tumor subtypes and molecular subtypes may associate varying sensitivity to therapies, validated predictive biomarkers are not available for clinical use [31].

## Local and locoregional disease

Surgery is the treatment of choice in stages I to III.

In ***tumors smaller than 7 cm*** (T1) the recommended treatment is partial nephrectomy (via open, laparoscopic or robot-assisted laparoscopic approaches), a technique that enables similar results to be achieved with better preservation of renal function [32]. Radical nephrectomy is an alternative if partial nephrectomy is not possible. Ablative procedures are options for elderly patients or those with high surgical risk, and in cases of multiple bilateral tumors, such as hereditary RCC, especially in small tumors. Renal biopsy is recommended if surgery is not possible [33]. Active surveillance is an option in elderly patients with significant comorbidities or short life expectancy and solid tumors < 4 cm [34].

In ***T2 tumors measuring* > *7 cm,*** laparoscopic radical nephrectomy is the treatment of choice, while open surgery is called for in ***T3 and T4 tumors***, albeit laparoscopic surgery can be contemplated in certain situations. Lymphadenectomy and suprarenalectomy are not indicated if there is no evidence of invasion on imaging tests(1), although the latter should be considered in upper pole tumors > 4 cm or > T3 [35].

The evidence regarding the treatment of venous thrombus is based on retrospective studies and poses a challenge not exempt of complications. Surgical intervention should be evaluated when feasible, as it may be associated with prolonged survival [36].

### Adjuvant treatment

Until recently, several studies with angiogenesis and mTOR inhibitors had failed to demonstrate an overall survival benefit [37–42]; however, only one of the studies (in which sunitinib (S-TRAC) was administered) had demonstrated a disease-free survival benefit.

More recently, the results of a phase III trial have been published (Keynote-564), in which 994 intermediate- or high-risk patients (pT2 tumors with grade 4 or sarcomatoid features; pT3, any grade, node-negative tumors; pT4, any grade, node-negative tumors; any pT, any grade, node-positive tumors, or stage M1 with NED (defined as resection of the primary tumor and solid, isolated, soft-tissue metastases) were randomized to receive pembrolizumab vs. placebo for 1 year [43]. A benefit in disease-free survival (primary endpoint of the study) was demonstrated at 24 months (77.3% vs. 68.1%; HR 0.68; 95% CI 0.53–0.87; *P* = 0.002). A non-statistically significant trend toward a benefit was seen in overall survival in the pembrolizumab arm (HR 0.54, 95% CI 0.30–0.96, *P* = 0.0164).

Neoadjuvant treatment should be deemed experimental, in as much as it has only been studied in small trials.

## Recommendations

- Partial nephrectomy is recommended in T1 tumors, as well as in bilateral tumors or in patients with only one functioning kidney. Level of evidence: I. Grade of recommendation A.
- Radical nephrectomy is recommended in T2-4 tumors. Level of evidence: II. Grade of recommendation: A.
- Treatment with adjuvant pembrolizumab is an option for intermediate- or high-risk patients, as well as after complete resection of oligometastatic disease. More data are required in the future, including positive overall survival data. Level of evidence: I. Grade of recommendation: C.
- Surgical intervention should be contemplated when feasible, as it may be associated with prolonged survival. Level of evidence: III.

## Clinical and genomic prognostic classifications

The Memorial Sloan Kettering Cancer Center classification was formerly used for cytokine therapy [44]. Currently, the International Metastatic RCC Database Consortium (IMDC), based on a cohort of 645 patients and subsequently validated, constitutes the gold-standard prognostic assessment model for systemic therapy for metastatic RCC. The IMDC system includes six validated prognostic factors: Time from diagnostic to systemic therapy < 1 year; Karnofsky performance status (PS) < 80%; hemoglobin level below the lower limit of normal; corrected calcium above the upper limit of normal; neutrophils above the upper limit of normal; platelets above the upper limit of normal. The model classifies patients as good (0 factors), intermediate (1–2 factors), and poor (3–6 factors) risk [45, 46]. It has been validated in various pivotal randomized trials and is also predictive in second and successive lines of treatment and in non-clear cell RCC [47].

PD-L1 status has been studied to determine both its prognostic and predictive role. A meta-analysis appeared to establish the negative prognostic value of higher levels of expression of PD-L1 [48]. As for its possible predictive role, contradictory results have been reported in two meta-analyses, including several first-line, immuno-oncology (IO) combinations randomized trials [49, 50]. Considering all information, PD-L1 is not ready for routine use in clinical practice.

In recent years, several molecular models have been studied with the aim of identifying a predictive value. Beuselinck et al. identified four molecular subgroups with different sensitivity to sunitinib. The ones that associated better outcomes were ccrcc2 and ccrcc3 [51]. These subtypes were tested in a prospective, phase II trial with interesting results. Several prospective trials with IO combinations have inspired the intention to identify predictive molecular subgroups. In IMmotion150, comparing atezolizumab/bevacizumab with atezolizumab or sunitinib and following atezolizumab or sunitinib, angiogenic and T-effector profiles correlated with different outcomes depending on therapy [52]. The JAVELIN Renal 101 trial compared avelumab + axitinib vs. sunitinib and the authors identified a 26-gene immune signature and an angiogenesis signature with a different survival benefit for each treatments [53]. Moreover, seven molecular subgroups were identified among the IMotion151 sample, proposing different activity profiles [54]. On the other hand, in the CheckMate 214 trial, an association between inflammatory genes and survival was reported for IO combination [55, 56]. Nevertheless, none of these can be considered to possess any prognostic value.

To date, other biomarkers in tumor tissue, such as tumor-infiltrating immune cells or tumor mutation burden, as well as circulating biomarkers, such as circulating DNA, soluble PD-L1, or cytokines and inflammatory markers, have failed to demonstrate a definitive prognostic or predictive role. In a recent communication, the presence of CD8 + T-cell tumor infiltration correlates with a lack of favorable PBRM1 mutations, suggesting that there is a correlation between immune phenotypes and somatic alterations [57].

Recommendations:

- Clinical prognostic classification, preferably IMDC, should be used for treatment decision-making in mRCC. Level of evidence: I. Grade of recommendation: B.
- Genomic classification, including PD-L1 study, should not be used as a prognostic or predictive tool for treatment decision-making in mRCC. Level of evidence: II. Grade of recommendation: C.

## Role of surgery in advanced renal cell carcinoma

The role of surgery in advanced disease is limited to cytoreductive nephrectomy, metastasectomy in oligometastatic disease, and palliative nephrectomy due to symptoms.

The role of cytoreductive nephrectomy has been modified based on improvements in systemic treatments.

At the time of interferon immunotherapy, two randomized studies comparing nephrectomy followed by interferon alpha *versus* interferon alpha established the role of upfront cytoreductive nephrectomy after demonstrating an overall survival benefit [58, 59].

At the time of antiangiogenic monotherapy, two randomized studies evaluated the role and timing of nephrectomy in advanced disease [60–62]. Using a non-inferiority design, the CARMENA study compared upfront nephrectomy followed by sunitinib with sunitinib alone in patients with intermediate and poor prognosis according to MSKCC criteria. The primary endpoint was median overall survival (18.4 months for sunitinib vs 13.9 months with surgery plus sunitinib; HR 0.89, 95% CI 0.71–1.10), thereby meeting the non-inferiority target (upper limit < 1.20)0.60 In a follow-up analysis, it appears that cytoreductive nephrectomy may be beneficial for patients with a single risk factor [61]. The SURTIME study sought to compare upfront nephrectomy followed by sunitinib and delayed nephrectomy after three cycles of sunitinib, but closed prematurely due to poor recruitment. The primary endpoint was progression-free survival for delayed nephrectomy (32 months vs 15 months), whereas one of the secondary endpoints was overall survival. However, the study’s poor accrual (fewer than 100 patients) meant that overall survival was the object of exploratory analysis [62]. After these two studies, upfront cytoreductive nephrectomy could be recommended for patients with 0–1 risk factors and good overall status, while those with ≥ 2 risk factors should be put on systemic treatment and nephrectomy should be reserved for those whose response to such treatment is very good.

Some retrospective studies have assessed the role of cytoreductive nephrectomy. Two of them, in particular, are particularly salient. The National Cancer Database and the IMDC. The first analyzed 15,390 patients identified in the National Cancer Database who were treated between 2006 and 2013 and revealed that overall survival was significantly better in nephrectomized patients (35%; 17.1 months vs 7.7 months) [63]. The second study relied on the IMDC to identify, 4639 patients eligible to participate in the study. It, too, concluded that there is an overall survival benefit for nephrectomized *versus* non-nephrectomized patients and that said benefit does not differ whether they receive antiangiogenic therapy or immunotherapy [64].

There are no prospective studies with current immunotherapy evaluating the role of nephrectomy; thus, physicians are recommended to follow the recommendations put forth during the era of antiangiogenic therapy while we await the results of the ongoing studies NORDIC-SUN, CYTOSHRINK, and PROBE.

As for metastasectomy in oligometastatic disease, there are different scenarios in which surgery can play a role, i.e., in subjects who present with synchronous oligometastatic (recommended when there are < 3 metastases), metachronous (disease-free interval > 1 year), or residual disease following a good response to systemic treatment. The strongest predictors of prolonged survival were a disease-free interval from nephrectomy to metastases > 1 year, ≤ 2 vs > 2 sites (especially if the lung was involved as opposed to the brain), ECOG 0–1, and absence of sarcomatoid features [65]. Different forms of radiotherapy can also be considered as local treatment in selected cases.

## Recommendations

- Debulking or cytoreductive nephrectomy should not be deemed mandatory in patients with intermediate-poor IMDC/MSKCC risk who require systemic therapy. Level of evidence: I. Grade of recommendation: A.
- Cytoreductive nephrectomy may play a role in the management of advanced renal cell carcinoma in individuals with limited metastatic burden amenable to surveillance or metastasectomy, in patients requiring palliation, and potentially delayed cytoreductive nephrectomy in patients with a favorable response or stable disease after initial systemic therapy. Level of Evidence: II. Grade of recommendation: B.
- Metastasectomy can be contemplated in selected patients having a limited number of metastases or long metachronous disease-free interval. Level of evidence: II. Grade or recommendation: C.

## First-line systemic therapy for metastatic clear cell RCC (mccRCC)

Metastatic ccRCC includes a variety of poorly understood clinical situations with different prognoses, including patients with indolent oligometastatic relapses, and others with very aggressive disease associated with short survival. On the other hand, most therapies available exert their effect by acting on the microenvironment instead of the tumor cells themselves.

For many decades, the only active treatments were non-specific immunotherapies (interferon and IL-2) that were associated with high toxicity and 10% long-term survival in a selected subgroup of patients [66].

Following the discovery that RCC is closely associated with the loss of VHL gene activity, with increased VGEF and growth factors, angiogenesis, and anaerobic metabolism, a number of antiangiogenic tyrosine-kinase inhibitors (TKI) against the endothelium VGEF receptor (VEGFR) were developed and examined in phase III trials. These drugs exhibited a statistically significant increase in PFS against interferon (sunitinib [67]), placebo (pazopanib [68, 69]), and sorafenib (tivozanib [70]). TKIs achieved a median PFS of approximately 8–11 months and a median OS approaching 2 years, becoming the standard first-line treatment for the overall population of patients with metastatic ccRCC. These results varied according to the prognostic subgroups described in the IMDC classification, which was specifically developed for patients with metastatic ccRCC treated with antiangiogenic TKIs or bevacizumab. TKIs were particularly effective for patients in the “good prognostic group”, who achieved a median OS of 43 months at a time when effective salvage treatments were not yet available [71]. Even in this subgroup, some authors recommend local treatment of metastases and/or close follow-up for selected, asymptomatic, well-informed patients with the oligometastatic disease having a small tumor burden [72]. Finally, another VEGFR-TKI, cabozantinib, improved ORR and PFS compared with sunitinib in a randomized phase II trial in subjects with intermediate and poor risk [73], while the mTOR inhibitor (temsirolimus) was associated with longer OS *versus* interferon in a population of patients having a poor prognosis [74].

Recently, immune check point inhibitors (ICIs) promoting antigen presentation (Ipilimumab) or blocking antiPDL1/PD1 signaling to reverse tumor immune evasion mechanisms (atezolizumab, avelumab, nivolumab or pembrolizumab) have been developed against RCC. Due to the poor results achieved with TKIs in the intermediate and poor IMDC subgroups, the Checmate-214 phase III trial compared the combination of Ipilimumab-Nivolumab versus sunitinib in these subgroups and detected higher ORR and OS with the combination therapy [75]. After a median follow-up of 42 months, the median OS was 47 vs 26.6 months (HR: 0.66) and ORR 42% (11% CR) vs 26% (2% CR) in favor of the combination of drugs; in addition, a plateau in PFS was observed at approximately 35% after 2 years in both study groups [76]. A post hoc analysis suggested that the OS benefit might persist in patients who discontinued therapy due to adverse side effects. After these results, the combination of Ipilimumab-Nivolumab was considered standard for patients with IMDC intermediate-poor prognosis. In an exploratory analysis, no specific benefit was seen in the good-risk population.

Several combinations of ICIs and TKI anti-VEGFR have been compared with sunitinib in phase III trials. The IMmotion 151 trial [77], demonstrated a 3.5-month increase in PFS with atezolizumab-bevacizumab in the PDL1 + subgroup, but not in OS in the overall population [78]. Likewise, no significant increase in OS has been reported in the JAVELIN renal 101 trial with avelumab-axitinib, despite increased PFS in both the PDL1 + treatment arm and the overall study population [79].

**Fig. 1:**
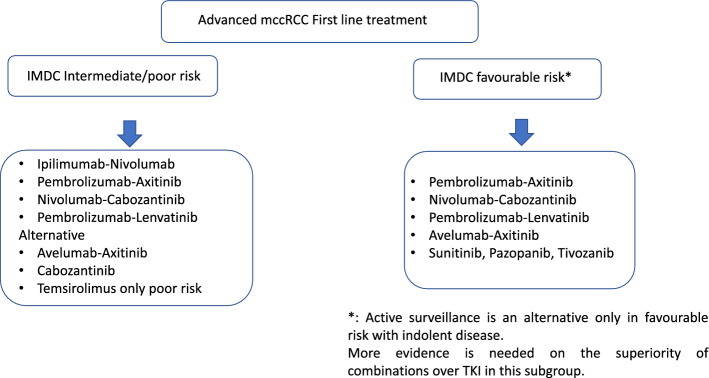
Advanced mccRCC First Line treatment. See text for levels of evidence

However, three trials have clearly demonstrated the statistically significant superiority of ICI-TKI combinations over sunitinib in ORR, PFS, and OS in the whole population of metastatic ccRCC. In the Keynote 426 study (pembrolizumab + axitinib vs sunitinib) [80], ORR was 59% vs 36%, median PFS 15.1 vs 11.1 months (HR 0.69), and median OS was not reached vs 35.7 months (HR: 0.53). In the CheckMate 9ER trial (nivolumab + cabozantinib vs sunitinib) [81], ORR was 56% vs 27%, median PFS 16.6 vs 8.3 months (HR: 0.51), and median OS was not reached in either group (HR: 0.60). Finally, the CLEAR trial (lenvatinib + pembrolizumab vs sunitinib) demonstrated an ORR of 71% vs 36%, with median PFS of 24 vs 9 months (HR: 0.39), and median OS was not reached in either group (HR: 0.66) [82]. All of these trials have consistently reported up to 70% grade 3 or higher adverse events of any cause both groups that are usually manageable, although as many as 5–20% of the participants discontinued of at least one of the drugs. Despite clear evidence of efficacy with the above combinations, certain areas of uncertainty persist. First, it should be noted that there are no definitive predictive factors allowing the medical oncologist to choose the most appropriate treatment for each patient. Second, indirect comparations among the ICI-TKI combinations are methodologically flawed given the differences in the baseline characteristics of the populations included in each trial. Third, all of these trials were designed to test hypothesis on the entire population of patients with metastatic ccRCC. Despite all the trials being stratified by IMDC or MSKCC prognostic classifications, subgroup analyses should be regarded as exploratory. More specifically, some uncertainty may arise with respect to the IMDC good prognosis subgroup, where none of the combinations obtained a clear advantage in terms of OS, despite the fact that some combinations displayed a higher ORR and longer PFS over sunitinib in the subgroup analysis. On the other hand, exploratory analyses of these trials (including Checkmate 214) invariably exhibited an advantage in OS over sunitinib in the subgroup with sarcomatoid differentiation (Fig. 1). Finally, an improvement in PFS has been reported recently with the triple combination of ipilimumab – nivolumab – cabozantinib vs sunitinib in the COSMIC-313 trial; nevertheless, OS results are still pending [83].

## Recommendations

Until predictive factors became reliable, the choice of first-line treatment for patients with metastatic ccRCC should be based on the local availability of approved drugs, patient comorbidities and prognosis, including the need for a quick response, as well as the design of a global therapeutic strategy with salvage options for subjects who do not respond or who relapse (Fig. 1).

Considering the whole population of patients with metastatic ccRCC:

- The combination of pembrolizumab + axitinib, nivolumab + cabozantinib, or pembrolizumab + lenvatinib can be considered the first options based on the benefit obtained in OS over sunitinib. Level of evidence: I. Grade of recommendation: A.
- Given its superiority on PFS over sunitinib, the combination of avelumab + axitinib is an alternative when other combinations are not available. Level of evidence: I. Grade of recommendation: B.
- Sunitinib, pazopanib, and tivozanib are reasonable options when the above-mentioned combinations are not available. Level of evidence: I. Grade of recommendation: B.

In the context of an individualized decision-making based on IMDC subgroups, in addition to the above recommendations:

- In patients with IMDC intermediate or poor prognosis, Ipilimumab + nivolumab should also be considered standard, given the benefit observed in OS over sunitinib. Level of evidence: I. Grade of recommendation: A. In this subpopulation, cabozantinib could be preferable to sunitinib based on the longer PFS obtained in a randomized phase II study. Level of evidence: I. Grade of recommendation: C Although not widely used, temsirolimus remains an option for poor-risk IMDC patients. Level of evidence: I. Grade of recommendation: C.
- No definitive evidence is available as to the benefit of the anti PD1/PDL1 plus either ipilimumab or TKI over sunitinib alone in the IMDC favourable subgroup. For asymptomatic patients with indolent and good-prognosis disease, active surveillance can be considered. Level of evidence: II. Grade of recommendation: C.

## Second-line treatment and sequences

In the past, the standard of care in first line was a TKI in monotherapy and many patients are still being treated with VEGF in monotherapy today. The anti-PD1 inhibitor nivolumab [84, 85] or the oral TKI targeting VEGFR, MET, and AXL VEGFR-TKI cabozantinib [86, 87] are the standard treatment for patients progressing to a previous anti-VEGF treatment. Both trials demonstrated increased OS and PFS as compared to everolimus. The combination of lenvatinib (another anti-VEGFR1-3, -FGFR, and -PDGFR oral TKI) with everolimus [88] exhibited improved PFS over everolimus in a randomized phase II trial in this population.

With the advent of first-line IO-based combinations, the therapeutic sequence in mRCC has changed and prospective randomized studies exploring what to do after failure of IO-based combinations are lacking. Furthermore, the phase III TIVO-3 trial [89] revealed greater PFS associated with tivozanib compared to sorafenib in a heavily pretreated population.

Several retrospective series have investigated the usefulness of multiple tyrosine kinase inhibitors (sunitinib, pazopanib, axitinib, cabozantinib, lenvatinib-everolimus) after IO treatment [90–100]. On the whole, these series displayed an ORR between 20–54% and PFS or TTF ranging from 6 to 13 months.

Prospective data exploring single-agent activity cabozantinib comes from a post hoc analysis of the METEOR trial. Of 32 patients who had received prior IO therapy (5% of the total cohort), activity favored cabozantinib over everolimus consistent with the overall population, median PFS (HR, 0.22), and a trend in overall survival with an ORR of 22% versus 0% [101]. A post hoc analysis from the TIVO-3 study, in which tivozanib was compared to sorafenib as third or fourth-line therapy in mRCC, also investigated the usefulness of tivozanib post-IO. Of 350 patients, 91 (26%) had previously received treatment with IO; median PFS favored tivozanib at 7.3 months compared to 5.1 months with sorafenib (hazard ratio 0.55). The overall response rate was not reported in this cohort.

Prospective data have also been published from small phase II trials with axitinib [102], cabozantinib [103], and sunitinib [104]. A phase II trial with dose escalation of axitinib was carried out in 40 patients who had received checkpoint inhibitor therapy as the most recent treatment. There was no limit on the number of previous therapies and 70% had received prior VEGF therapy. The trial reported 8.8 month PFS and an ORR of 45% [102]. In the Breakpoint trial, 22 patients were treated with cabozantinib after adjuvant or first-line PD-1/PD-L1-based therapy (as monotherapy or in combination). The primary endpoint was PFS, which was 9.3 months and the ORR was 43% [103]. In the Immunosun trial, subjects who had progressed following a first-line regimen consisting of an ICI-based therapy were treated with sunitinib. PFS and ORR were 5.6 months and 19%, respectively [104].

## Recommendations

- In patients with advanced RCC previously treated with one or two antiangiogenic tyrosine-kinase inhibitors, nivolumab, and cabozantinib are the recommended options. Level of evidence: I. Grade of recommendation: A. Decisions to use either agent may be based on the expected toxicity and on contraindications for each drug, as randomized data is lacking.
- Axitinib, everolimus, lenvatinib + everolimus, and tivozanib are alternatives for second-line, providing that they are available and patients cannot receive nivolumab or cabozantinib. Level of evidence: I. Grade of recommendation: B. In addition, they may also be acceptable options following nivolumab and cabozantinib. Level of evidence: III. Grade of recommendation: C.
- For patients who progress after initial immunotherapy-based treatment, we suggest treatment with an anti-VEGFR TKI. Options include cabozantinib, axitinib, tivozanib, sunitinib, and pazopanib. Further research is required in this context. Level of evidence: III. Grade of recommendation: C.
- Patients should be encouraged to participate in clinical trials whenever possible.

## Non-clear renal cell renal carcinoma.

Approximately, 15–20% of renal cell carcinomas (RCC) are classified as non-clear cell renal cell carcinoma (nccRCC), which are then further divided into multiple distinct subtypes based on histological and molecular characteristics. Subtypes of nccRCC include papillary, chromophobe, collecting duct, renal medullary, and translocation RCC, which account for 10–15%, 5–7%, 1–2%, < 1%, and < 1% of all RCCs, respectively [105]. All subtypes can have sarcomatoid differentiation. Median survival of individuals with localized nccRCC varies with histology, with more favorable outcomes in patients with papillary and chromophobe RCC. In the metastatic setting, however, survival in all subtypes of nccRCC is uniformly worse compared to ccRCC, due to the inherent aggressiveness of these cancers and a lack of effective systemic treatment options [106]. These patients have significantly lower RR and poorer mPFS and mOS than those with ccRCC (ORR 10.5%, mPFS 7.4 months, and mOS 13.4 months) [107]. Clinical data are limited in these rare histological subtypes, which tend to be excluded from controlled phase III trials, and most antitumor activity data are derived from retrospective studies, expanded access programmes, and prospective single-arm studies. The treatment of localized forms (stages I, II, and III) is comparable to ccRCC. There is no evidence of the efficacy of adjuvant treatment, as the majority of trials in the adjuvant setting did not include patients with nccRCC, and only two of them included a number too small as to enable any conclusions to be drawn. The role of cytoreduction in advanced disease is controversial, given that the CARMENA and SURTIME trials did not include nccRCC patients. Two retrospective series, the International Metastatic Database Consortium (IMDC) and the National Cancer Database (NCD) included 510 and 3201 patients, respectively, with advanced nccRCC tumors. Both showed a significant impact on OS in favor of the arm that included surgery (20.6 *vs.* 9.6 and 17.1 *vs.* 7.7 months, respectively) [107, 108]. Regarding metastasectomy, nccRCC patients are scarcely reflected in clinical trials and subgroup analyses are not available. Therefore, cytoreductive nephrectomy and/or metastasectomy would be an option in some patients, and the decision must be made on a case-by-case basis. The first prospective data in advanced disease were collected from a subset analysis of the phase III study of temsirolimus, which allowed non-clear-cell renal cell carcinoma and exhibited comparable efficacy with the clear-cell renal cell carcinoma cohort [109]. Three subsequent randomized phase II trials comparing sunitinib to everolimus included a diverse array of non-clear histologies and provided evidence that front-line sunitinib induces better PFS, albeit to a modest degree, when compared with PFS observed in clear-cell disease [110–112]. These results were further supported by data from expanded access programs. A meta-analysis was performed that included 365 patients with advanced nccRCC. The pooled HR for PFS was 1.30 (95% CI 0.91–1.86, *p* = 0.15), indicating a trend toward superiority of sunitinib over everolimus, although the results failed to reach statistical significance [113]. Other anti-VEGFR tyrosine kinase inhibitors, such as axitinib and pazopanib, have been evaluated in small prospective series and retrospective analyses that have yielded promising activity comparable to that achieved in the clear-cell population [114–116]. Nevertheless, most of these studies only patients enrolled subjects with papillary and chromophobe tumors. A small retrospective study and real-world data from an Italian expanded access program have reported moderate efficacy with cabozantinib, revealing overall response rates of 23%, mPFS of 8 months, and mOS of 12 months [117, 118]. As the majority of the papillary-specific studies investigated the use of c-MET inhibition, due to the increased incidence of alterations in the MET proto-oncogene in these tumors [119], cMET inhibitors such as cabozantinib appear to represent an acceptable option instead of the usual anti-VEGF TKIs. The safety and efficacy of immune checkpoint inhibitors (ICIs) have been explored in nccRCC in the KEYNOTE-427 study of pembrolizumab, a subgroup analysis of the CheckMate 374 study of nivolumab, and an expanded access program for nivolumab [120–122]. Additionally, a phase II trial of atezolizumab and bevacizumab included patients with nccRCC and clear cell renal cell carcinoma with sarcomatoid differentiation (sccRCC) [123]. Most of the patients had papillary RCC. ORRs ranged from 13 to 26%. Data from prospective studies with combinations of ICIs and TKIs are also available. In the CALYPSO phase I/II trial utilizing the combination of savolitinib + durvalumab, an ORR of 29% was achieved in 41 patients with papillary renal cell carcinoma (up to 40% in MET-driven tumors) [124, 125]. Cabozantinib and nivolumab have displayed activity as well in 47 nccRCC patients with different histologies, with an ORR of 47.5% in papillary, unclassified, and translocation-associated RCC. In addition to these data, sarcomatoid histologies, tumors of the collecting ducts, and medullary renal carcinoma have traditionally been contemplated as benefitting from chemotherapy. Two series present their role in sarcomatoid histology, yielding a modest benefit in these patients. Some data suggest that sarcomatoid tumors are highly inflamed tumors that usually associate poor-risk features and are sensitive to ICIs. Nevertheless, there are no randomized clinical trials available to confirm the efficacy of immunotherapy in first-line treatment [126, 127]. Collecting-duct tumors tend to be resistant to systemic therapy. Nonetheless, platinum-based chemotherapy is usually recommended based on small prospective and retrospective series. After first-line therapy, there are insufficient data to allow any recommendation to be made. However, at least for papillary tumors, which are the most common non-ccRCCs, the use of the ccRCC algorithm is an acceptable option.

## Recommendations

- Clinical data are limited in nccRCC, which are usually excluded from controlled phase III trials. Therefore, enrolment into specific clinical trials is strongly recommended. Level of evidence: V. Grade of evidence: A.
- There are no available data regarding post-nephrectomy adjuvant treatment in localized nccRCC.
- In the first-line setting, the most robust data exist for sunitinib, although other targeted therapies, such as TKI and mTOR have limited data. While specific data are not available, the choice of treatment should be based on each specific subtype:
  - **Papillary**: Sunitinib: Level of evidence: II. Grade of evidence: B. Pazopanib: Level of evidence: III. Grade of evidence: C. Everolimus: Level of evidence: II. Grade of evidence: C. Cabozantinib: Level of evidence: IV. Grade of evidence: c.
  - **Cromophobe**: Sunitinib: Level of evidence: II. Grade of evidence: C. Pazopanib: Level of evidence: III. Grade of evidence: C. Everolimus: Level of evidence: II. Grade of evidence: C.
  - **Collecting duct/Medullary**: Cisplatin or carboplatin- based regimen: Level of evidence: III. Grade of evidence: C.
  - **Sarcomatoid**: Sunitinib. Level of evidence: II. Grade of evidence: B. Pazopanib: Level of evidence: III. Grade of evidence: C. Nivolumab+ipilimumab: Level of evidence: IV. Grade of evidence: C.
- After first-line, no recommendation is possible based on available data.

## Methodology

This guideline has been developed based on the consensus of ten genitourinary medical oncologists, designed by the Spanish Society of Medical Oncology (SEOM) and the Spanish Oncology Genitourinary Group (SOGUG), and an external review panel comprising two experts designated by SEOM. The Infectious Diseases Society of America–US Public Health Service Grading System for Ranking Recommendations in Clinical Guidelines has been used to assign levels of evidence and grades of recommendation (Table 4).

**Table 4:** Levels of evidence and grades of recommendation

Category, grade	Criteria
**Quality of evidence**	
I	Evidence from at least 1 properly randomized, controlled trial*
II	Evidence from at least 1 well-designed clinical trial without randomization, from cohort or case-controlled analytical studies (preferably from more than 1 center), or from multiple time series or dramatic results from uncontrolled experiments
III	Evidence from opinions of respected authorities based on clinical experience, descriptive studies, or reports of expert committees
**Strength of recommendation**	
A	Both strong evidence of efficacy and substantial clinical benefit support recommendation for use. Should always be offered
B	Moderate evidence of efficacy, or strong evidence of efficacy but only limited clinical benefit, supports recommendation for use Should generally be offered
C	Evidence of efficacy is insufficient to support a recommendation for or against use, or evidence of efficacy might not outweigh adverse consequences (e.g., drug toxicity, drug interactions) or the cost of chemoprophylaxis or alternative approaches Optional
D	Moderate evidence of lack of efficacy or of adverse outcome supports a recommendation against use Should generally not be offered
E	Good evidence of lack of efficacy or of adverse outcome supports a recommendation against use Should never be offered

*Although not included in the original table, systematic reviews and meta-analysis of well-designed randomized clinical trials have also been considered as the level of evidence I

## Data Availability

Not applicable.
